# Beyond the Brachial Plexus: Reapplication of the Thoracic Paravertebral Block for Upper Arm Fistula Surgery

**DOI:** 10.7759/cureus.42863

**Published:** 2023-08-02

**Authors:** James C Krakowski, Daniel T Le, Stuart A Grant

**Affiliations:** 1 Department of Anesthesiology, University of North Carolina at Chapel Hill School of Medicine, Chapel Hill, USA; 2 Department of Anesthesiology, Naval Medical Center San Diego, San Diego, USA

**Keywords:** clinical case report, upper limb surgery, arterio-venous fistula, regional anesthesiology, thoracic paravertebral block, supraclavicular brachial plexus block

## Abstract

Patients requiring complex upper arm arteriovenous fistulas or grafts may not be suitable candidates for a single regional anesthesia technique and monitored anesthesia care because the necessary thoracic (T2) dermatomal area of the medial, upper arm remains spared by any solitary brachial plexus (C5-T1) technique. An infiltrative intercostobrachial nerve block can often be used in conjunction with a brachial plexus block; however, coverage may still be incomplete. This case report describes the use of a high thoracic paravertebral block in conjunction with a brachial plexus block to achieve adequate anesthetic coverage for an upper arm arteriovenous fistula creation procedure extending into the axilla. The result of this technique showed adequate coverage of the upper arm and demonstrates that paravertebral blocks are a reasonable adjunct for proximal upper arm arteriovenous fistula procedures.

## Introduction

For patients undergoing arteriovenous (AV) fistula surgery, the most common and advantageous form of anesthesia includes the use of regional anesthetic nerve blocks [1]. A benefit of regional anesthesia via peripheral nerve blocks (PNBs) for AV fistula procedures arises from their sympatholytic effects on vascular flow [2]. Previous studies have specifically shown that the use of PNBs for AV fistula procedures increases arterial blood flow and venous diameter, both of which decrease rates of thrombosis and fistula failure [2]. Regional anesthesia may also decrease hospital length of stay following AV fistula surgery [3]. However, the use of regional anesthesia can be limited based on the location of the desired surgical site. Patients requiring complex upper arm AV fistulas or grafts often may not be suitable candidates for surgeon-administered local anesthesia or brachial plexus block alone due to the multiple nerves supplying the upper arm, including the intercostobrachial nerve (ICBN) that does not originate from the brachial plexus.

Because the ICBN originates from the T2 nerve root below the C5-T1 nerve roots comprising the brachial plexus, brachial plexus blocks are ideally supplemented with an ICBN infiltration block for medial, upper arm coverage [4,5]. Unfortunately, the ICBN block performed subcutaneously may only achieve 50-90% adequate coverage [6], likely stemming from significant anatomic variability [7].

In this report, we describe a combined brachial plexus block and high thoracic paravertebral block (PVB) to achieve a complete sensory block of the upper arm in a patient undergoing AV fistula placement. The thoracic PVB is a dose-dependent block placed within the paravertebral space lateral to the vertebral column where the spinal nerves exit the intervertebral foramina [8].

## Case presentation

A 49-year-old female with a past medical history of elevated body mass index (34.9 kg/m2), asthma, obstructive sleep apnea, hypertension, orthopnea, paroxysmal nocturnal dyspnea, seizures, focal segmental glomerulosclerosis, and end-stage renal disease requiring hemodialysis presented for the creation of a new, left-sided AV fistula due to failure of a previous left brachiocephalic fistula created four months prior. Her medications included gabapentin and acetaminophen for lower back pain. On preoperative examination, the patient was found to be 157.5 cm in height, weighing 86.5 kg, and possessed a well-healed surgical scar site of her otherwise unremarkable left upper extremity. The patient’s preoperative lab work was unremarkable except for an elevated serum creatinine value of 3.26 mg/dL. Previously, the patient received a preoperative supraclavicular brachial plexus nerve block (SCB) in conjunction with intraoperative total intravenous anesthesia (TIVA) via propofol infusion with spontaneous, unassisted ventilation throughout the case without issue. Based on ultrasonographic examination prior to this case, the planned procedure necessitated a proximal surgical site in the upper arm approximating the axilla. Due to the location of the surgical site, there was concern that a brachial plexus block combined with an ICBN block would not provide adequate coverage to the proximal extent of the surgical site. Instead, the decision was made to perform a high thoracic-level PVB in conjunction with a SCB to achieve adequate surgical depth coverage suitable for intraoperative TIVA sedation via propofol infusion with spontaneous ventilation.

**Figure 1:**
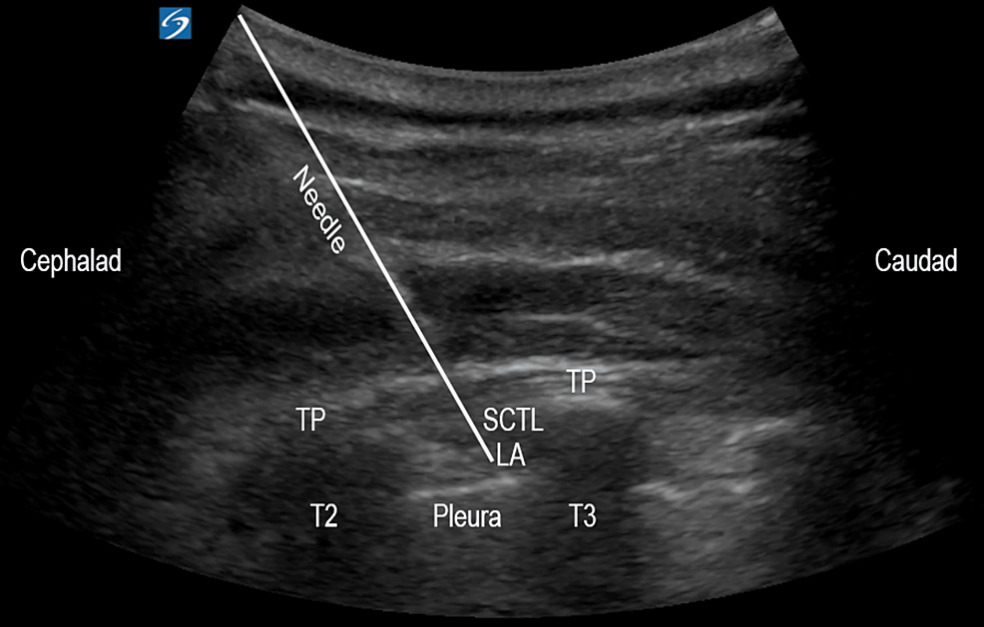
Left-sided, ultrasound-guided thoracic paravertebral block via a parasagittal, in-plane approach at the T2-3 interspace. TP: Transverse process, SCTL: Superior costotransverse ligament, LA: Local anesthetic.

A T2-3 interspace thoracic paravertebral block was selected to provide dermatomal coverage extending into the axilla that would otherwise be spared via a brachial plexus (C5-T1) block alone. Following informed consent, the patient was placed in a seated position for the left-sided, thoracic PVB. A time-out procedure was performed, and the patient subsequently received 2 mg of intravenous midazolam for anxiolysis. A curved array probe (2-5 MHz C60xp probe, Sonosite, Bothwell, WA) and 21-gauge four-inch needle were used to deposit 10 mL of 0.5% ropivacaine within the left T2-3 interspace using a parasagittal view (Figure 1).

**Figure 2:**
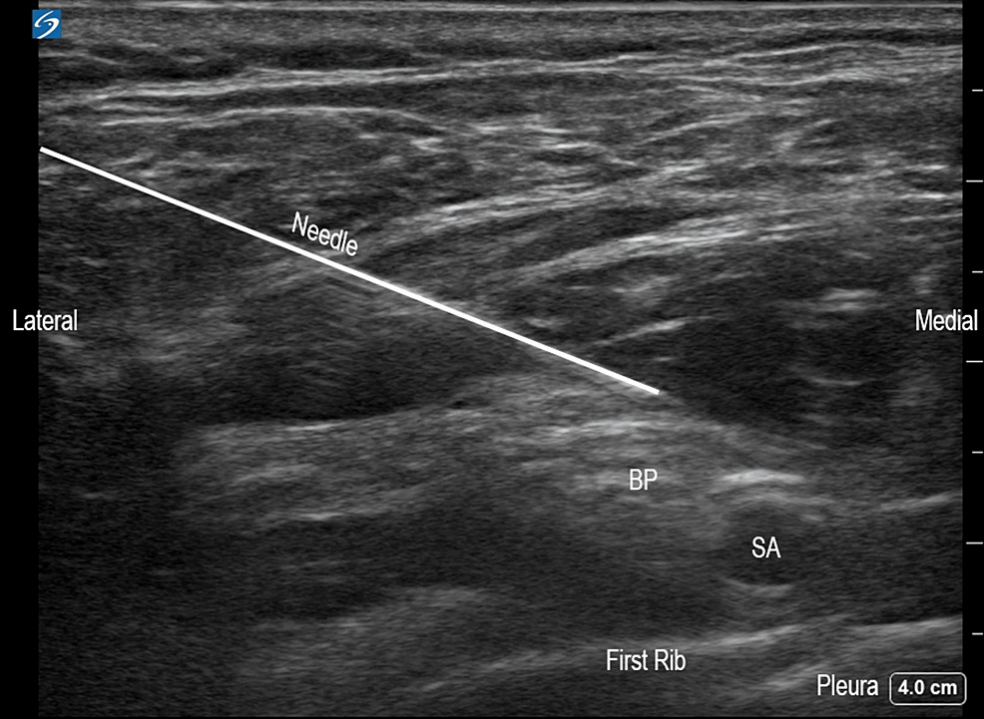
Left-sided, ultrasound-guided supraclavicular approach to the brachial plexus block. BP: Brachial plexus, SA: Subclavian artery.

A SCB was next chosen to provide brachial plexus coverage of the upper arm extending distal to the lower arm. The patient was thus placed in a semi-seated position for the performance of a left-sided SCB. A linear probe (6-15MHz HFL50xp probe, Sonosite, Bothwell, WA) and a 21-gauge four-inch needle were used to deposit 20 mL of 0.5% ropivacaine surrounding the left brachial plexus via a supraclavicular approach (Figure 2).

**Figure 3:**
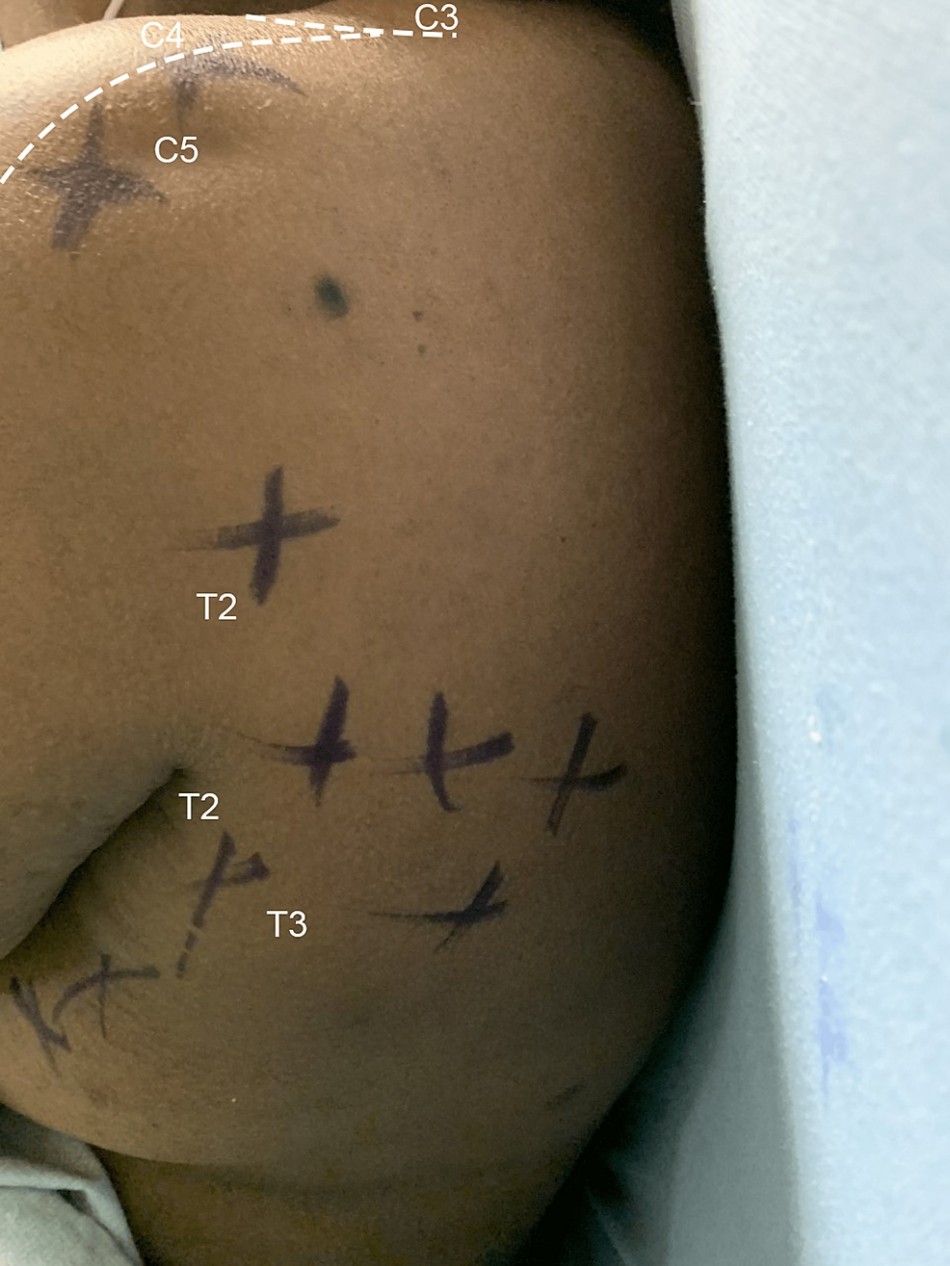
Distribution of coverage (purple skin marker) using ice testing at 10 minutes following the left-sided thoracic paravertebral block and supraclavicular block combination. Both C5 (brachial plexus) and T2-3 thoracic dermatomal coverage are shown.

**Figure 4:**
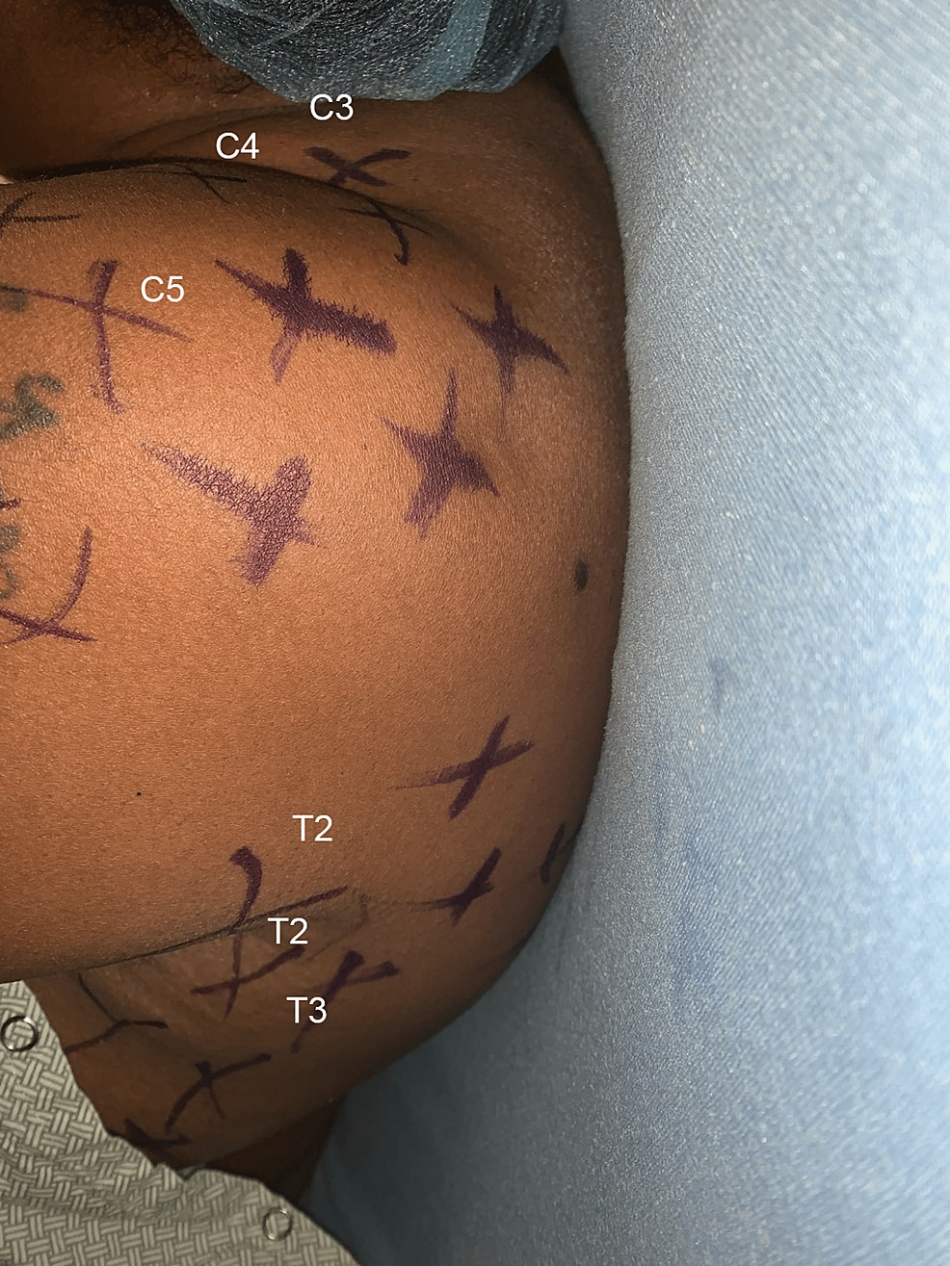
Distribution of coverage (purple skin marker) using ice testing at 30 minutes following the left-sided thoracic paravertebral block and supraclavicular block combination. Expanded C5 (brachial plexus) proximal to distal spread and T2-3 posterior to anterior spread are shown.

For each block, an in-plane needle approach was utilized, negative aspiration was confirmed, and small aliquots (3-5 mL) of local anesthetic were administered. Both procedures were tolerated well by the patient without complications. Ice testing was performed 10 minutes after block placement, revealing loss of cold sensation in the T2-3 dermatomal distribution (Figure 3). After 30 minutes, the dermatomal coverage of the block expanded (Figure 4).

Throughout the intraoperative period, the patient was successfully maintained with a propofol infusion (50-100 mcg/kg/min) for anesthetic maintenance. Oxygen was delivered via a simple face mask at 6L/min, and oxygen saturation was maintained above 99% via spontaneous ventilation throughout the case. Vital signs remained stable with transient phenylephrine infusion (0.3-0.6 mcg/kg/min) administered for hemodynamic support following initiation of the propofol infusion and stopped prior to emergence. After the procedure, the patient recovered in the post-anesthesia care unit (PACU) and maintained a numeric pain rating score of zero out of ten without additional analgesics. She was discharged from the PACU directly to home the same day. Post-operative follow-up revealed a gradual return of motor and sensory function within 48 hours without complication.

## Discussion

The inclusion of regional anesthesia offers numerous benefits for AV fistula surgery including improved vascular flow, decreased fistula failure rate, and decreased hospital length of stay [1-3]. Anesthetic coverage via a brachial plexus block alone is often inadequate, particularly when the surgical procedure extends into the proximal axilla. Although inclusion of an ICBN (T2) covers the T2 dermatomal distribution outside of the brachial plexus (C5-T1) [4,5], the ICBN performed subcutaneously may only achieve 50-90% adequate coverage with significant anatomic variability [6,7]. A high thoracic PVB can mitigate this weakness by supplying coverage of the necessary T1-2 dermatomes. We report a case of successful regional anesthesia via combined SCB and thoracic PVB for creation of a proximal AV fistula. The addition of the PVB successfully achieved upper medial arm coverage extending into the ipsilateral axilla (see Figures 3 and 4) that would otherwise be spared through use of a brachial plexus block alone.

The local anesthetic volumes of 20 mL of 0.5% ropivacaine for the SCB and 10 mL of 0.5% ropivacaine for the thoracic PVB were chosen to both minimize the risk of local anesthetic systemic toxicity and promote effective surgical anesthesia. A combined volume of 30 mL of 0.5% ropivacaine represented a total calculated dose (150 mg) based upon 3 mg⋅kg−1 maximum dosing of the patient’s lean body weight [9]. For the SCB injection, 20 mL of local anesthetic volume was selected to increase the likelihood of surgical anesthesia within a recommended volume range of 20-25 mL [10,11]. Although the thoracic PVB may be performed with larger volumes of local anesthetic, a smaller volume of 3-4 mL per thoracic level has been described as superior to ensure reliable dermatomal spread [12]. Because of the relatively narrow dermatomal band (T2) needed to anesthetize the medial, upper arm, successful coverage via a T2-3 thoracic PVB was achieved with a relatively lower local anesthetic volume of 10 mL and confirmed via ice testing.

Although both the SCB and thoracic PVB are considered low-risk procedures, the performance of each block creates the potential for complications. Both peripheral nerve blocks may cause bleeding with hematoma formation, infection, pain at the site of injection, local anesthetic systemic toxicity, or failure to achieve the desired anesthetic coverage. Performance of the SCB is often accompanied by ipsilateral phrenic nerve palsy that may cause up to a 30% reduction in respiratory mechanics [10]. Caution should be taken when performing the thoracic PVB due to a 0.5% risk of pneumothorax [12]. Hypotension may also occur following the thoracic PVB due to the proximity and potential spread to the epidural space [12,13]. However, significant hypotension in a normovolemic patient following the thoracic PVB remains a rare occurrence in contrast to thoracic epidural anesthesia [12,13]. Risk factors for hypotension following a thoracic PVB include bilateral block performance, using a medially directed needle, and the injection of local anesthetic volumes greater than 25-30 mL at a single level [12]. We acknowledge that the thoracic PVB is technically a more challenging block to perform versus the ICBN infiltration in conjunction with a more significant side effect profile. Fortunately, the increased use of ultrasound guidance has provided a greater understanding of thoracic PVB efficacy and safety [12].

Although intraoperative hypotension was observed for this patient requiring transient phenylephrine infusion, we believe the etiology was most likely related to the combination of intravenous propofol and midazolam. In order to minimize epidural spread, the thoracic PVB was performed unilaterally using an ultrasound-guided parasagittal, in-plane approach with a small volume of local anesthetic injection. Both the intravenous bolus and infusion of propofol have been shown to cause hypotension via peripheral vasodilation, particularly when combined with benzodiazepine therapy [14,15]. Furthermore, the transient nature of the hypotension with immediate resolution following the discontinuation of the propofol infusion also implicates the intravenous anesthetic rather than the regional anesthetic techniques performed.

We recognize one limitation of this case is that a single case is inadequate to assess the rate of intra-operative success of achieving adequate coverage when using this combination of blocks, particularly when compared to an intercostobrachial nerve block. We also recognize that we are unable to assess this specific block combination with regard to long-term AV fistula patency in comparison to the intercostobrachial block. To the authors' best knowledge, no studies have been done comparing the PVB to an intercostobrachial block when used in conjunction with a brachial plexus block. Furthermore, no studies have been done investigating the effectiveness of the PVB on fistula hemodynamics and long-term patency. Therefore, it is difficult to conclude if one technique is superior to the other.

Future research is still needed to assess the non-inferiority of this combination of PNBs compared to the traditional ICBN block technique in both the adequacy of surgical coverage and long-term AV fistula patency. Of note, there is currently a clinical trial being conducted exploring this question [16]. The aim of that study is to compare the PVB versus the ICBN block in providing optimal conditions for patients undergoing upper arm AV surgery.

## Conclusions

In this study, we describe the supplementation of a brachial plexus block with a high thoracic (T2-3) PVB rather than the more traditional ICBN block for a patient undergoing an AV fistula creation procedure. This case is significant because it presents one alternative strategy to effectively anesthetize the proximal upper extremity for AV fistula surgery. Further research is needed to determine the effectiveness and safety of combining the thoracic PVB and brachial plexus block versus other regional anesthetic techniques prior to recommending a definitive change in practice.
